# The Galvanic Current

**Published:** 1897-02

**Authors:** H. G. Husted

**Affiliations:** Oberlin, Ohio


### The Galvanic Current.

BY H. G. HUSTED, OBERLIN, OHIO.

Read before the Ohio State Dental Society, December 3, 1896.

It is my object in this paper to give a description of the galvanic cell, its construction, action, and some of its practical applications to dentistry. It is very evident that electricity is to take a prominent place in the operating-room and laboratory of the up-to-date dentist. What has been accomplished in the past few months is, to my mind, but the beginning of still greater results to follow, and these greater results are to be brought about by each one who has any light to give upon the subject, although it may be but a little—freely contributing to the general fund.

Galvanism is that form of electricity which is generated by chemical action. The galvanic cell is a development of the voltaic pile, which, as originally constructed, consisted of pairs of zinc and copper plates, between which pieces of blotting-paper or flannel, moistened with an acid or saline solution, were placed, from which an action was set up, whose strength was proportional to the number of pairs used; the current was the result of the action of the acid upon the zinc plates.

The most familiar form of the galvanic cell consists of a vessel containing diluted sulphuric acid or a solution of sal-ammoniac, in which a plate of zinc and a plate of carbon are partially immersed, the carbon surface being much greater than that of the zinc.

If the zinc is pure, there is no action as long as there is no external connection between the two, but if they are connected by a wire the acid attacks the zinc, and electricity is generated, which passes through the fluid to the carbon within the cell, and from the carbon to the zinc over the wire outside the cell.

The question is in every one's mind, "What is this thing which manifests itself in such a wonderful manner?"

An expert says: "It is a question no man can answer." One definition given is, "The name given to the unknown thing, matter or force, or both, which is the cause of electrical phenomenon."

The street-car motorman, who calls it "juice," understands its real nature as well as the wisest expert. (The zinc is the generating plate, and the carbon the collecting plate.)

There are many substances which may be used besides those mentioned; whatever combination is used, one of them must be more readily acted upon by the acid than the other. The generating-plate will show loss by dissolution, while the other will show little or no loss.

The plates are also called positive and negative, the carbon being the positive from the fact that the electric current, outside the cell, first manifests itself at that point and passes to the zinc—

there is no variation of this order when these elements are used. There are conditions when this may appear to be untrue. Our battery fluid is all right; the zinc and carbon properly connected, and yet there is no current—the battery has become polarized. When the battery is in action there is a decomposition of the water into its elements, oxygen and hydrogen. These gases show polarity the same as metals, oxygen being negative and hydrogen positive.

We are speaking now of the action within the cell, and must consider the zinc as positive and the carbon as negative. The negative oxygen is attracted to the electro-positive zinc and unites with it, forming the oxyde of zinc; the positive hydrogen collects on the electro-negative carbon. There being no action between them, the hydrogen bubbles remain upon and cover the surface of the carbon. The battery fluid attacks hydrogen more readily than the zinc, and the latter becomes the collecting plate, thus reversing the current. This goes on as long as there are hydrogen bubbles upon the carbon. These may be removed by simply opening the current or raising the carbon out of the fluid for an instant.

Impurities in the zinc, such as particles of iron or arsenic, will also interfere with the action of the battery, causing a local action upon the zinc. This is avoided by having the zinc coated with a film of quicksilver.

Any substance over which a current of electricity passes freely is said to be a good conductor. Copper being such, is commonly used for this purpose. The larger the conductor the greater the amount of current which will pass over it in a given time. The conductivity of any conductor depends upon the area of a cross-section. The opposite of conduction is resistance. By reducing the size of a conductor the amount of current will correspondingly be reduced.

That property by which electricity is enabled to overcome resistance is called electro-motive force, the unit of measurement being the volt.

The electro-motive force of a cell is not determined by the size of the cell. A cell the size of a common tumbler will have the same electro-motive force as one the size of a buccket, when composed of the same elements.

Zinc and carbon are commonly used because of the great difference of electric level or potential which they furnish.

The electro-motive force of a battery may be increased by increasing the number of cells. The quantity of current produced by a cell depends upon the size of the generating-plate. A cell having a generating surface of one hundred square inches will give five times as great a current as one with twenty square inches, because there will be five times as great a consumption of zinc. The quantity of current may also be increased by increasing the number of cells. The unit of measurement of quantity is the ampere.

We now come to that part of our subject which, to me, is intensely interesting—the quantity arrangement and the potential arrangement of cells. There are certain purposes for which a current of large quantity is required, such as producing cautery and other heating purposes. It has already been shown that the quantity of current depends upon the surface of zinc exposed to the action of the fluid.

A cell with a zinc plate 4 by 5 inches would have a surface of forty square inches. If much larger, the cells would be cumbersome, especially if a surface of two hundred square inches was required. But five cells, whose aggregate surface would be two hundred square inches, could be joined in such a manner that the same result would be produced. By joining the zincs in all the cells together, and the carbons together, we would have the same amount of surface acted upon by the fluid, and the same amount of zinc consumed as in the one large cell, and the quantity of the current would be the same. The electro-motive force is the same in the small cell as in the large one, and when the five cells are connected in this manner the electro-motive force still remains the same as in one cell—about one and five-tenths volts.

In connecting for potential there is no advantage in having large cells, in fact, the smaller the better, within certain limits. Let us take the same five small cells and connect the zinc of the first cell with the carbon of the second, and the zinc of the second with the carbon of the third, etc. We will then have the carbon of the first cell and the zinc of the last cell free; to these we connect our terminal wires. What is the result? We have a current with an electro-motive force five times as great as in the former combination. Each cell added raises the potential of the series, and the electro-motive force is increased as many fold as there are cells in the series.

The resistance of the human body is very great, and it is the electro-motive force of the current that overcomes that resistance. Ten large cells would not send a greater current through the body than an equal number of small cells of like construction. For cataphoric purposes, the number of cells needed varies greatly, from six to ten cells being sufficient, in the majority of cases, when under proper control.

The dry cell, in common use, has the same elements—zinc and carbon—as described, but the exciting agent is prepared in the form of paste, and the cell is sealed up. This makes a very convenient form for our use, as the battery can be kept within a small compass. But some form of the Leclanche cell will be found more durable. These cell are intended for use only where there is considerable resistance, or the time they are in use is short. They are soon exhausted if left long at a time with closed circuit.

Ohm's Law, which is the foundation of all electrical measurements, is as follows: "The strength of the current, passing through any part of the circuit, varies directly as the difference of the potential between the elements, and inversely as the resistance in the circuit itself." The formula of Ohm's law is that the current equals the electro-motive force divided by the resistance.

$$C=\frac{E}{R}$$

We now come to a practical application of the galvanic cell in relieving the pain when preparing cavities in the teeth for filling.

I do not propose to speak of what has been accomplished within the past year, because you are all as well, or better, informed upon the subject than I am. I propose, rather, to give you somewhat in detail an account of what I have been trying to do, and some of the results.

Three years ago a friend who was somewhat interested in the subject of electricity asked the privilege of using a back-room in my office for experimental purposes. His apparatus was somewhat crude, consisting of a half dozen copper cans about six inches square and three inches deep, some small flower-pots for porous cups, and common sheet-zinc for plates. I had no knowledge whatever of the subject, but I watched him with a good deal of interest, although nothing whatever resulted from his work, which was very soon abandoned, all his cans, etc., being left where he used them, the copper sulphate creeping over the edges of the cans and on to the floor making considerable of a muss.

Later in the winter I spent a little time occasionally in trying my hand at it, my object being to produce a current that would run a small motor. My outfit consisted of the copper cans which my friend had left, which were partly filled with the copper sulphate solution, into which I suspended sheets of zinc. The result was a violent local action and very small external current.

In January or February, 1894, I had a lady-patient who wished me to fill a cavity in the superior left labial incisor, labial surface. Upon attempting to excavate the cavity after adjusting the dam, I found it so sensitive that it was impossible to proceed. After trying all the remedies I could think of, without any good effect, I determined to try electricity, such as I had. I think now that I must have had the cells connected two in series and three in parallel, which would have given an electro-motive force of about three volts. I connected one wire to a piece of tin and applied to the side of the face ; the other was adjusted to cervical clamp on the tooth. There was no sensation experienced from the current, but I could use the engine-burr or excavator without a particle of pain to the patient. To make sure that there was no make-believe on her part, without her knowledge of what I was about to do, I several times disconnected the wire from the clamp. In every instance the pain of excavating was as great as at the first. Upon connecting again, it was perfectly insensible to any amount of cutting. This gave me the assurance that electricity, properly applied, would do the work. Subsequent trials were more or less successful, but none equal to the first. I had no way of testing the current used, and, in fact, I had little idea of the nature of the current used. I began to study the problem, but could get no light from any source. I tried various kinds of batteries, such as I could construct myself, large and small, few and many cells, connected in all manner of combinations, but with little success. I next turned my attention to the subject of resistance, and constructed rheostats of various kinds. Not having any appliance for measuring a current, it was impossible to do any accurate work. A few dollars invested in a McIntosh milliampere meter and a work on "Electro-Therapeutics" proved to be a good investment. In the chapter on the "Production of Cutaneous Anæsthesia," in which the action of cocaine with the galvanic current was described, I made this note on the margin. "Try on tooth." But, being more interested in finding the action of a direct current, I did not push my investigation in that direction.

When the success of the electro-cocaine process was announced last spring, the appliance which I already had was adaptable to that purpose, and excellent results were accomplished from the first.

The chief objection to cataphoresis, then, as now, was that too much time was required in the operation. If it were impossible to accomplish the same result in some other way, this objection ought not to be a valid one. The operator who is unwilling to take a few minutes extra time in order to avoid actual suffering on the part of his patient is not worthy of that patient's patronage.

When we learn how to control the direct current so that it can be successfully used in the major part of our operations we shall have made a great advance over cataphoresis.

There are certain conditions which it is necessary to observe in attempting the electrotonic method. First, the current used must not be so great as to cause pain of itself. Second, the engine burr must be fine cut and sharp. Third, the pressure must be light and the engine run rapidly. The full strength of one, and sometimes two cells, may be used without the patient feeling any unpleasant sensation. Then, again, a quantity of resistance must be introduced into the circuit from one cell in order to make it so the patient will not feel it. The amount of current required will vary in different teeth of the same individual. In case the cavity is shallow, the caries not having advanced very far, a greater amount will usually be required than where the tooth-structure is badly broken down. Yet, no set of rules will apply in all cases. One must learn from experience about what is needed, and then try until he obtains the best results. I have often tested the current used on the milliampere meter and have found it to vary from one-tenth to four or five milliamperes, sometimes even more under a pressure of from one and a half to four and a half volts.

In cases where the cavity is located on the labial surface of the incisor teeth, adjust the dam, using a cervical clamp. Place the negative electrode on the side of the face as in cataphoresis, and attach the positive electrode to the clamp, as previously described. This will allow the use of other instruments beside the engine. Or the positive electrode may be attached to some point on the engine so that there will be a metallic circuit to the burr. Special pains must be taken to avoid making a circuit through the operator. The safer way is to have the hand-piece insulated with thin rubber tubing.

Until the electrotonic method has become more fully developed a large percent of cases may prove partial or total failures, but if one has an appliance which can be adapted to either method it is but the work of a moment to shift the positive electrode from the engine to the tooth and use the electro-cocaine process.

The very pertinent question might be asked, What is the physiological effect of a galvanic current upon a nerve?

One writer says that a current traversing a certain length of nerve divides it into two sections or zones which physiologically differ. That portion nearest the negative pole has its irritability increased, and its condition is called catelectrotonous. That portion near the negative pole has its irritability decreased, and its condition is called atelectrotonous, and that the point of difference between these two conditions depends upon:

First. The size of the electrodes.

Second. The distance they are apart.

Third. The electro-motive force of the current.

Fourth. The length of time the application is continued.

This principle can be successfully applied in the case of ordinary tooth-ache from an exposed nerve by using a mild current for a few minutes.

I have used the Galvanic battery in cases of soreness of the face or threatened suppuration with good results, the negative pole being applied to the sore portion with a large electrode, and the positive held in the hand, using as strong a current as possible without causing pain and of fifteen or twenty minutes duration, repeated two or three times.

---

The discussion on the foregoing four papers on Cataphoresis will appear in the next issue.

